# Clinical psychologists’ perceptions of telephone consultation for low-income patients during the COVID-19 pandemic: an interview study

**DOI:** 10.1017/S1463423624000495

**Published:** 2024-10-31

**Authors:** Ségolène Payan, Pablo G. Barbosa Bergami, Xanthie Vlachopoulou, Pascale Baligand, Jean-Christophe Peronnet, Marion Robin, Aziz Essadek

**Affiliations:** 1 Eos Psy, Paris, France; 2 Institut de Recherche et de Formation àl’Action Sociale de l’Essonne, Évry-Courcouronnes, France; 3 Département de Psychiatrie de l’Adolescent et du Jeune Adulte, L’Institut Mutualiste Montsouris, Paris, France; 4 Institut de Psychologie, Université Paris Descartes, Paris, France; 5 INSERM U1123 Epidémiologie Clinique et Évaluation Économique Appliquées Aux Populations Vulnérables, Paris, France; 6 EA4432 Laboratoire de Psychologie de l’Interaction et des Relations Intersubjectives (INTERPSY), Nancy, France; 7 Department of Adolescent and Young Adult Psychiatry, Institut Mutualiste Montsouris, Paris, France; 8 Hopitaux Saint-Maurice, Saint-Maurice, France

**Keywords:** clinical psychology, COVID-19, precariousness, social housing, telemental health

## Abstract

**Aims:**

We will examine the processes of change in psychological practice that have been altered by the lockdown.

**Background:**

During the COVID-19 pandemic, low-income populations, notably residents of social homes or shelters, were shown to be particularly susceptible to contagion. During lockdown, telephone-based psychological consultations became the norm.

**Methods:**

In this qualitative research, we carried out semi-structured interviews with 10 psychologists working in social homes or shelters. Interviews were transcribed verbatim. Data were studied using consensus qualitative research.

**Findings:**

During lockdown, participants felt that isolation increased while medical and social support decreased. Psychologists had to adapt their work methods and work more closely with on-site staffs. After lockdown, there was an increase in mental health issues. Participants perceived that telephone consulting seemed to facilitate access to psychological help. Although psychologists have quickly adapted, a decrease in the quality of clinical work was a general assessment. Results stress the necessity to train French psychologists in telemental health practices.

## Introduction

### Low-income patients during the pandemic

In the context of the COVID-19 pandemic, low-income, precarious^1^ populations, and especially those at the very bottom of the social strata, were shown to be more susceptible to contract the disease than the general population (Lewer *et al*., 2020) but were also more likely to experience a deterioration in their mental health (Scarlett *et al*., 2021; Essadek *et al*., 2022, 2023). Prevalence was particularly high for people living in social houses or homeless shelters (Roederer *et al*., 2021). This had direct repercussions on the activities of clinical psychologists working in such places.

### Psychologists’ work in the field

Mental health interventions in these contexts are usually based on a proactive approach, grounded in the patients’ environment (Auffret, 2016; Mercuel, 2018), aimed at promoting access to care, as well as its continuity, for people who are somewhat excluded from it (Krawitz & Watson, 1997), often due to the loss of social objects inherent to social exclusion and precariousness (Spira, 2017; de Ruffi & Zdanowicz, 2018). To prevent, identify, and take into account the psychosocial suffering specific to precariousness, as well as its possible consequences (Furtos, 2008, 2009; Rönnblad *et al*., 2019; Utzet *et al*., 2020), psychologists establish their settings as close as possible to where people live while still respecting their privacy (Sorba, 2018). They work to restore confidence in the interpersonal and social bond (Tap, 2004) by adopting an accessible clinical stance that favours informal conversation; they also seek to be the interface between the residents and the professionals who manage the housing facilities, as well as health and social services (Arveiller & Mercuel, 2011). This work is necessary because precarious patients suffering from depression do not consult a practitioner (Rondet *et al*., 2015).

### Context of the COVID-19 pandemic

The pandemic and the application of containment protocols, notably the establishment of the first lockdown (March 17 to May 11, 2020, in France), made it impossible to carry out psychological interventions along these lines in social housing facilities and shelters. The use of the telephone therapy/services/etc. became the norm. Meeting patients ‘face-to-face’, whether in an office, in their apartments, or through informal on-site discussions, was no longer feasible. Communication with the different managing staffs present on site (when they were present) or with neighbouring psychiatric and medico-social institutions also had to be done remotely. In addition, health risks were a source of anxiety for both residents and workers (Probst *et al*., 2020). Unusual situations or new problems have emerged, while old issues were often aggravated (Torales *et al*., 2020). In light of these changes, it seemed relevant to question the way in which the proximity and interface work usually carried out by psychologists in these contexts was affected and to explore the experience and the perceptions of psychologists of such matters. The goal of this research was to shed light on the specificities of remote consultation work carried out by psychologists with low-income individuals and to foster reflection at an institutional and transversal level (Wahlbeck *et al*., 2017) that might surpass and frame individual practices deployed urgently and spontaneously to cope with the onset of the pandemic. So, we will examine the processes of change in psychological practice that have been altered by the lockdown.

## Methods

### Study design and conception

This qualitative study was designed according to the COREQ-32 guidelines (Tong *et al*., 2007). This approach was chosen because it is the only reporting guidance for qualitative research to have received other than isolated endorsement (Booth *et al*., 2014). It follows a qualitative exploratory descriptive design based on semi-structured interviews and consensus qualitative research (Hill *et al*., 2005). Similar methods were used in the context of similar research related to the experience of teleconsultation by patients during the COVID-19 pandemic (Lockard *et al*., 2022; Pogorzelska *et al*., 2023). The interviews concerned the experiences of psychologists regarding the change in practices during the first period of lockdown and the ensuing months. This study has been approved by the University of Lorraine and is registered under the number 2021-154.

### Participants and recruitment

Using a purposive sampling method, 10 psychologists who work in social homes or shelter facilities were selected. Due to the risk of contagion, all of the research interviews were carried out by video conference. Subjects were previously contacted and invited to participate in the study. They were made aware of the goals of the study and gave their informed consent in writing prior to the interviews. Inclusion criteria were (1) being a clinical psychologist intervening in social homes or homeless^2^ shelters and (2) being a volunteer to participate in the study. Sample size was mainly determined by practical reasons; that is, it comprised almost the totality of a psychological team readily accessible to the research, and it also corresponded to an amount of data deemed processable by a research team with limited resources.

### Data collection

The framework of the interview was drawn up with reference to relevant literature on the subject through the work of an expert committee, made up of two professionals from the area and also two researchers with experience on the topic. The final interview guide included seven open-ended questions probing various aspects of the experience of psychologists during lockdown and afterwards. The questions are listed in Table 1. All interviews were conducted in October 2020, seven months after the implementation of the lockdown that necessitated changes in professional practices.

**Table 1.** Questions asked to psychologists in order to collect their perceptions

Question 1	Did you experience any change in the issues patients talked about?
Question 2	How did you experience the changes in consultation modalities?
Question 3	Was there an impact on your practices?
Question 4	Do you find any advantages or disadvantages for your clinical practice in these modifications?
Question 5	Have they affected your therapeutic relationship with residents?*
Question 6	Has your post-lockdown practice changed?
Question 7	How did you manage these modifications to the framework and the management of the therapeutic relationship?

*Since the clinical activity concerned here does not take place within mental health institutions but in housing facilities, we shall favour the term ‘resident’ over ‘patient’.

### Data analysis

The interviews were recorded and transcribed verbatim. Transcriptions were then analysed according to the recommendations (Hill *et al*., 2005). To accomplish this, initially, two members of the research team organized the information in the interview transcripts into domains and core ideas. This encoding was then verified and corrected by a third member of the team, serving as auditor, in order to minimize interpretation bias. The domains and core ideas were then reviewed, in line with the consensus process, in research meetings attended by all researchers (with the exception of the interviewer). In these meetings, the cross-analysis leading to the establishment of general categories organising the data was also carried out. Finally, representativeness of thematic categories was determined via inter-case comparative analysis by the researchers, who had first derived the domains and core ideas from the transcripts. Thus, themes might be considered ‘general’ (mentioned by all the respondents, or all -1), ‘typical’ (mentioned by more than half of the participants), ’variant’ (mentioned by a number of participants between three and half the sample), or ‘rare (mentioned by less than three participants) (see Table 2).

**Table 2.** Psychologists’ perception about residents’ experience and psychologists’ experience

	Residents’ experience	Psychologists’ experience
During lock down	Emergence of new psychological problems***	Changing the work tool*** ● Using the telephone*** ● Using *WhatsApp*
	Intensification of isolation**	No use of visioconsultation*** ● Residents not equipped/skilled** ● Protection of psychologists’ privacy
	Decrease in medical and social support*	Improvement in the collaboration with on-site staff* Reduced collaboration with the network and partners*
After lock down	Increase in anxieties* Increase in decompensations* Increased malaise*	Improvement in the collaboration with on-site staff** Apprehension concerning a potential emotional backlash from professionals Relief* Resuming face-to-face consultations Increase in the number of residents seen* Continued use of the telephone*
Perception of the telephone as a tool in clinical practice	Adaptation (patients)*** Using the telephone simpler for residents who hadn’t previously met the psychologist*	Adaptation (professionals) *** ● Need to establish a new organizational setting for work** ● Need to participate in team meetings to avoid feeling overwhelmed* Positive perception of remote consultation during lockdown*** ● Facilitates access to psychological consultations*** ● Preserves therapeutic bond** ● Overcoming presuppositions regarding tele consultation Negative impact on the quality of clinical work*** ● Lack of non-verbal cues* ● Teleconsultation less effective than face- to-face for low-income, precarious individuals**

‘General’ themes are indicated by ***, ‘typical’ are indicated by **, ‘variant’ are indicated by *, and ‘rare’ have no specific markings.

### Preunderstanding

The research team is composed of three men and three women. All of the authors are psychologists with a PhD in the field, with the exception of the penultimate author, who is a master’s degree student in psychology. They all have qualitative research experience. The first two authors worked and contributed at the same level in this research. They, together with the third and the last author, contacted the subjects and organized the interviews. The same four authors each have more than 10 years of practice in this sector. For this reason and to increase the reliability of our study, following the recommendation of Levitt *et al*. (2017), a different researcher conducted the interviews. The first two authors did the initial encoding of the data. The third author carried out a literature review focused on the theme. The fourth author conducted the interviews with psychologists. The fifth author transcribed the interviews, and the last author is behind the conceptualisation of the study; he also served as auditor for the encoding of the interview data. All the authors participated in the development of the research project and the conceptualisation of the interview guide.

During the initial meetings, authors discussed their subjective stances on the research subject. Though all of them had a fairly positive attitude towards telemental health, only one of them had very limited experience with it.

## Results

The contents of the interviews were organized in three main domains: ‘during lockdown’, ‘after lockdown’, and ‘perception of the telephone as a tool in clinical practice’. These domains were further divided into two categories. The first category concerned the psychologists’ perception of their own experiences; the second category concerned their view of the experience of the people they work with. Core ideas, which we also named themes, were then categorized within these domains according to their frequency of occurrence in the interviews.

Table 2 presents these results. Table 3 presents a brief socio-professional characterization of our sample.

**Table 3.** Socio-demographic variable

	Gender	Age	Years of experience	Therapeutic approach
1	Female	68	7	Psychodynamic
2	Male	30	7	Psychodynamic
3	Female	38	13	Psychodynamic
4	Female	36	8	Psychodynamic
5	Female	38	12	Systemic
6	Male	30	1	Psychodynamic
7	Female	39	14	Psychodynamic
8	Male	42	15	Psychodynamic
9	Female	41	15	Psychodynamic
10	Female	28	2	Psychodynamic

In the following sections, only ‘general’ and ‘typical’ themes in each domain will be analysed. Note that all quotes below are taken from the original interviews and have been translated from the original.

### During lockdown

#### Concerning the residents’ experience as perceived by the psychologists

##### Intensification of isolation

Regarding the psychologists’ perception of residents’ experience during lockdown, all psychologists noted an intensification of previous difficulties and notably isolation. The drastic reduction of social contacts, a global experience during this time, seems to have affected social housing residents even more intensely. That seems to be a consequence of the fact that much of their social interactions, before the pandemic, were those they established with social workers or health professionals. Indeed, many of them have health problems that require regular care from paramedical professionals, in particular nurses. Lockdown limited the available time and frequency of passage of many of these professionals, leading to an intensification of the isolation of residents. During lockdown, ‘the nurses spent less time (with the residents), and they took less time to communicate with the patients’.

##### Emergence of new psychological problems

Psychologists also perceived residents to experience other psychological problems such as stress, fear, and anxiety more intensely than before, or arising as a reaction to lockdown. According to the psychologists interviewed, ‘during lockdown, there was anxiety or even anxio-depressive syndromes linked to the situation’, and ‘it (lockdown) was rather anxiety-inducing, especially in the beginning’.

#### Concerning the psychologist’s experience

##### Changing the work tool

The change in working methods is unsurprisingly central to the experience of psychologists during this time. This could be characterized as ‘the transition from [being in] the presence of the other to the telephone’. Almost all of the interviewees used ‘only the phone’ for consultations. Indeed, psychologists indicate they ‘really switched entirely to the regular phone call’. If some practitioners perceived this change towards the exclusive use of the telephone in their work as ‘a form of continuity in support’, others think that ‘with the telephone, there was too much distance […this] prevented a certain number of issues from being addressed, particularly questions surrounding addiction’.

##### No use of videoconference

Most psychologists consider this decision as essentially practical, since most residents did not have access to a computer or telephone device equipped with a camera or did not have the skills to use video conferencing tools. ‘[These] people don’t necessarily have very advanced technologies’ on their phones.

It is interesting to consider the change of work tool vis-a-vis the perception of an intensification of the isolation of residents. ‘In fact, many people in a precarious situation don’t really use the phone, or use it very little’. As the telephone therefore almost entirely replaced face-to-face contacts, for many residents, this equated to a brutal reduction of all social contact, thus reinforcing their isolation. This was particularly felt by psychologists trying unsuccessfully to reach residents. ‘When you can’t reach someone because the number is wrong… These are people who change phones regularly. So the [therapeutic] bond is cut, can be cut, or has been cut in a fairly regular and repeated fashion’.

### After lockdown

#### Concerning the residents’ experience as perceived by the psychologists

There are no recurring ideas regarding residents’ experience after confinement. Though some psychologists tend to perceive an aggravation of psychological problems, there is no general trend, and perceptions vary. This variability seemed important to mention since it is the only domain for which no theme emerges that is ‘general’ or ‘typical’.

#### Concerning the psychologist’s experience

##### Improvement in the collaboration with on-site staff

The improvement in collaboration with the management of establishments, which for some seemed to develop during lockdown, continued and became a trend, perceived by more psychologists after the first lockdown: ‘There is now a more regular exchange of emails. The staff does not hesitate to send me emails even outside of my working hours, to anticipate and prepare my next visit, to exchange information’. The majority of psychologists interviewed explained that they had devoted more time to discussion with professionals who seemed to need support to better understand the psychological issues affecting residents following lockdown. In general, they relate more frequent contacts and a feeling that their input is more valued.

### Perception of the telephone as a tool in clinical practice

#### Concerning the residents’ experience as perceived by the psychologists

##### Necessary adaptation

Residents also had to make an adaptation effort, according to psychologists. Some residents were ‘a bit like a drowned man hanging on to his buoy’, without really knowing how to use it. A number of people in precarious situations did not have phones before the lockdown. They had to buy one or borrow one from the housing facilities in order to be able to be contacted by professionals in the medico-social sector. Residents were not always familiar with all the features of their phones.

#### Concerning the psychologist’s experience

##### Adaptation to teleconsultation

All the psychologists explained that they needed to adapt to the teleconsultation and that it was a considerable effort.

##### Need to establish a new organisational setting for work

A fundamental aspect of this category were the efforts to establish a new work setting. All the psychologists interviewed mentioned the organisational requirements of having had to adapt to telemental health methods. They evoke the need to organize a suitable working environment, a ‘necessary organization at home between private life and professional life’, because calling or receiving a phone call from home can lead to a feeling of intrusion into his/her personal life. Most of them could not establish precise work schedules at home, and many noted the abuses that this could lead to: ‘it never stopped!’. The patients did not respect the working hours of the psychologists; they called at all hours.

##### Positive perception of remote consultation during lockdown

Psychologists unanimously perceive the experience as a positive one. The main ideas that are at the foundation of this positive perception are as follows:

##### Facilitates access to psychological consultations

Participants consider telephone consultation has facilitated access to psychological help, especially for residents who use the phone intensively in their daily lives. ‘Having people on the phone allowed me to have access to some of them more easily than when I was present on site’. They felt that answering the phone may seem less demanding than ‘having to move around, to be confronted with otherness, which actually required a lot more (effort from the residents)’.

##### Preserves therapeutic bond

The psychologists interviewed also noted the usefulness of the telephone for maintaining therapeutic relationships. ‘The telephone is a tool that has made it possible to maintain a link’. Avoiding a total lack of contact during lockdown, teleconsultations allowed the continuity necessary for the therapeutic support of patients.

##### Negative impact on the quality of clinical work

###### Teleconsultation less effective than face-to-face for low-income, precarious individuals

In contrast, most psychologists have found that the clinical work done in this way is of lower quality compared to face-to-face meetings. ‘The fact of not seeing the person, and all the more with people who do not always know how to express themselves well or express what they feel (verbally) is a little complicated’. Psychologists have also found that it is more difficult ‘to de-mystify what the psychologist is on the phone than in a face-to-face situation’; telephone consultations could therefore be perceived as facilitating a ‘degradation of the bond, of the relationship’ even if the psychologists explain having tried to position themselves in ‘a form of continuity in the support’.

## Discussion

The social changes the pandemic entails will likely turn remote consultation into a common tool for clinical psychologists everywhere. The recent increase in the number of ‘hotlines’ providing psychological support in France, some of them managed directly by the hospital system, attests to the current importance of such practices. Understanding psychologists’ perceptions of this (relatively) new clinical practice seems paramount in order to efficiently integrate it into clinical activity. Therefore, in this section, we shall focus almost exclusively on the data regarding the perceptions psychologists have of the changes in their practice.

First and foremost, a globally positive tendency emerges from the interviews: despite the abrupt change of method and the adaptive effort it required, psychologists found the experience enriching. They appreciate the possibilities it opens for reaching people who, for one reason or another, do not have access to in-person psychological help; they also value electronic communication as a supportive tool aiding the maintenance of a therapeutic bond in extraordinary conditions. Psychologists display a willingness to adapt and globally perceive telephone consultation as a useful clinical tool. In this regard, our study is in line with global trends (Connolly *et al*., 2020).

It seems, however, important to delve deeper into the perceived downsides of such a tool. Common to all interviews was the perception of an unofficial increase in working hours. This difficulty seems inherent to remote work (Planchard & Velagic, 2020), and, in the present case, it is likely to stem mainly from the psychologists themselves being unable to limit their availability periods. Some clearly indicated that they took calls outside of their working hours. This apparently simple organisational matter is not a negligible factor, insofar as these excesses can constitute a major factor of professional stress and dissatisfaction, which may lead psychologists to turn away from telemental health practices.

It therefore seems that an important measure to facilitate the integration of remote consultations in the context explored here would be to clearly set the time frame for this kind of work and to scrupulously respect it. The subjects of this study suggest it themselves. This principle should be paramount as much for the psychologists as for the structures that employ them in order to reduce the cognitive and emotional load put on professionals.

This organisational blind spot is symptomatic of an underlying factor that seems central to understanding psychologists’ perception of remote consultation in the present context: the lack of preparation for this type of clinical activity. If psychologists overworked or let working hours mix indifferently with their domestic daily lives, it may be argued that it is mainly due to the absence of a predefined framework for this kind of remote consultation, whether it be on the organisational level or on the relational plane.

This lack of preparation and unfamiliarity with telemental health practices show in the interviews. None of the participants had previously worked in such fashion, nor did they have any specific training to do it.

Furthermore, the participants brought up some important issues in relation to financial barriers within the psychologists’ specific patient population, for example, not having access to phones and/or phones with cameras. This experience also confronted professionals with the social inequalities of the patients with whom they work. Indeed, teleconsultation cannot be made if there remain inequalities in access to technology (Cotton and Gupta, 2004; DiMaggio *et al*., 2004). Moreover, the use of teleconsultation depends on the usability of the information system (Dünnebeil *et al*., 2012), which is strictly connected to the perception we have of it (Altmann *et al*., 2022).

In that regard, they seem to be representative of the French context, where the integration of remote consultations into the skill set of psychologists and into the general offer of psychological care is still developing. Training and counselling in the field of ‘e-psychiatry’ and telemental health has actually boomed in the 2010’s, as Verpeaux (2016) indicates. But these practices remain at an initial stage of integration into the clinical skill set of French therapists (Massé *et al*., 2006; Haddouk & Schneider, 2020) by comparison to other national contexts. For instance, ‘telehealth’, in the broad sense, was only given a legal framework in France in 2010 (Decree No. 2010-1229 of October 19, 2010), and such practices were only included in the funding criteria of the French public health care system in 2018.

Psychologists in our study seem thus to have been confronted with fundamental questions regarding the use of remote psychological consultation and had to try to figure out the answers by themselves. This aspect also seems important in understanding that which is at the centre of the negative dimension of psychologists’ assessments of their experience with remote consultations: the overall perception of an impoverishment of the clinical work.

They tended to think of this reduction in clinical «richness» and efficiency, partially as a consequence of the lack of non-verbal cues necessary for a better grasp of the affective tonality of conversations. They also felt that the spontaneous calls made by the psychologists in the absence of a prior request expressed by the residents – a measure deployed during lockdown – could also have played a role in the aforementioned impoverishment. Likewise, some have mentioned the impossibility, in this context, of countering resistance (there is no interpretation possible when someone turns off their telephone). This could be read, on a more conceptual level, as a feeling of reduction of the possibilities of intervening on the relationship due to the media through which the relationship is established.

It seems reasonable to hypothesize that part of the difficulties encountered may be closely linked to the favoured means of communication. As mentioned, only the telephone was used for the remote consultations we studied. Video communication tools would probably have made it possible to overcome some (but not all) of the perceived hindrances, in particular, the absence of infra-verbal cues. It is necessary to highlight the specificity of this clinic (teleconsultation), in which technical aspects directly affect the construction of the therapeutic relationship (Scharff, 2012; Mathieu-Fritz, 2018).

And here, again, the lack of preparation and training seems to be a fundamental underlying factor. The psychologists interviewed were not previously trained to adapt to this specificity nor to efficiently use electronic means of communication in their practice, which is likely to have indeed rendered their clinical interactions less efficient, something of which they were conscious.

## Limitation

Though we have argued that, given the stage of development of telemental health in France, these perceptions might be representative of a much more general trend, it is necessary to stress the limitations of the present study. At the inclusion criteria, there was no limit on how long the psychologists had been a practitioner for. One had only been working for one year; therefore, their comparison to pre-pandemic work is pretty limited. We are also aware that data saturation was not likely achieved, and sampling size was not enlarged mainly due to limited research resources. During the period this research was carried out, many professionals were no longer connected to their professional contact details. Also, we were only able to reach a limited number of practitioners to participate in this study. Finally, our research interviews were carried out by video conference. This may have influenced our findings. Additionally, within the framework of consensual qualitative research, Hill *et al*. (2005) suggest having the results analysed by another research team, which we have not done. In summary, although our study provides significant insights into the experiences of psychologists, several additional limitations must be considered. The transferability of our results is restricted by the specific geographical context of the study and the institutional particularities in which the psychologists operate. Moreover, despite explicitly stating our positionality as researchers in this study, our stance may have influenced the data collected, despite our reflexive efforts to minimize this impact. Finally, the relevance of our results may be limited to similar environments and might not be applicable to other contexts.

We believe nonetheless in this study’s heuristic value, in view of the scarce data on this subject matter concerning the French context, and notably on psychologists’ perceptions of remote consultation. We hope it is a contribution that not only encourages further research concerning psychologists’ perceptions and attitudes towards telemental health practices, especially in France, but that its findings shed light on the importance of preparing professionals and defining the specific theoretical and practical framework within which it should function.

Given the present development of telemental health, our study advises caution. Though it underlines psychologists’ willingness to adapt, it also emphasizes the need to familiarize them with these practices and to establish a clinical framework allowing them to use these tools to their fullest potential. It also points to the perceived limitations of such practices. In this regard, the general perception of a reduction in therapeutic efficiency must also be viewed in the light of one final factor concerning the population with whom participants worked.

## Conclusion

The «digital divide» is a major factor in the dynamics of social exclusion in our time. The practical impossibility psychologists in this study encountered in using more advanced electronic communication tools with residents of social homes is an empirical occurrence of this social problem. Participants in this research have indeed resumed their standard modes of practice while still utilising the telephone simply as an aide in the care of precarious individuals. None of them envision the inclusion of teleconsultation in their daily work unless an exceptional period demands it again. They suggest that the social isolation in which many of those individuals are may be reinforced by digital means of communication from which they are excluded. Thus, they point to the iatrogenic potential of such practices in a precarious social context.

In a time when a certain enthusiasm for telemental health seems to develop, it is important not to neglect this aspect in psychological interventions dedicated to the more vulnerable elements within our societies.

## Data Availability

The datasets generated and/or analysed during the current study are not publicly available due [to data protection of participants] but are available from the corresponding author on reasonable request.

## Footnotes

1 ‘Precarious’ and ‘precariousness’ (‘*précarité*’) are often used in French to describe, beyond the economic and concrete living conditions, the existential insecurity of low-income populations. The works of Furtos (2008, 2009) define it clearly. Though this use of the word is less common in English, we decided to keep it here in reference to this field of research.

2 Psychologists in this study work in four types of facilities. These provide either temporary shelter for the homeless (*Centre d’Hébergement d’Urgence*, *Centre d’Hébergement et Réhabilitation Sociale*), ranging from a single night to a few months, or very low-rent, state-subsidized apartments for excluded and precarious individuals (*Résidences sociales* and *Pensions de famille*). Their time of presence in each of these facilities ranges from 6 hours a week to 3 hours every 2 weeks.
